# Intraspinal and Intracranial Neurotuberculosis, Clinical and Imaging Characteristics and Outcomes in Hospitalized Patients: A Cohort Study (2000–2022)

**DOI:** 10.3390/jcm12134533

**Published:** 2023-07-06

**Authors:** Ana Luisa Corona-Nakamura, Martha Judith Arias-Merino, María Guadalupe Miranda-Novales, David Nava-Jiménez, Juan Antonio Delgado-Vázquez, Rafael Bustos-Mora, Aldo Guadalupe Cisneros-Aréchiga, José Francisco Aguayo-Villaseñor, Martha Rocio Hernández-Preciado, Mario Alberto Mireles-Ramírez

**Affiliations:** 1 High Specialty Medical Unit, Western National Medical Center of the Mexican Institute of Social Security, Guadalajara 44340, Mexico; 27.correo@gmail.com (D.N.-J.); drjadv@hotmail.com (J.A.D.-V.); dr.rafaelbustos@gmail.com (R.B.-M.); aldogca@gmail.com (A.G.C.-A.); adicofer@hotmail.com (J.F.A.-V.); mrociohp@hotmail.com (M.R.H.-P.); 2 Western Clinical Research Institute, Zapopan 45030, Mexico; marthaariasmerino@gmail.com; 3 Analysis and Synthesis of the Evidence Research Unit, National Medical Center, XXI Century (IMSS), Mexico City 06720, Mexico; mmirandanovales@gmail.com; 4 Department of Philosophical and Methodological Disciplines, University Health Sciences Center, University of Guadalajara, Guadalajara 44340, Mexico

**Keywords:** intraspinal, intracranial, neurotuberculosis, clinical characteristics, imaging, outcomes

## Abstract

Neurotuberculosis (neuroTB) is a devastating disease, and is difficult to diagnose. The aim of this study was to analyze the clinical and imaging characteristics, and outcomes of a retrospective cohort (2000–2022) of hospitalized patients diagnosed with intraspinal and intracranial neuroTB. This work was designed through clinical, laboratory and imaging findings. Variables included: demographic data, history of tuberculosis, neurological complications, comorbidities and outcomes. Morbi-mortality risk factors were identified by univariate analysis. The cohort included: 103 patients with intraspinal and 82 with intracranial neuroTB. During the study period, in-hospital mortality of 3% for intraspinal and 29.6% for intracranial neuroTB was estimated. Motor deficit was found in all patients with intraspinal neuroTB. Risk factors for the unfavorable outcome of patients with intraspinal neuroTB were: age ≥ 40 years, diabetes mellitus (DM), diagnostic delay, kyphosis and spondylodiscitis ≥ 3 levels of involvement. Among the patients with intracranial neuroTB, 79/82 (96.3%) had meningitis and 22 patients had HIV infection (10 of them died). Risk factors for mortality from intracranial neuroTB were: HIV infection, hydrocephalus, stroke, lymphopenia and disseminated and gastrointestinal TB. Patients with intraspinal neuroTB had a significant number of destroyed vertebrae that determined their neurological deficit status. The mortality burden in intracranial neuroTB was conditioned by HIV infection and renal transplantation patients.

## 1. Introduction

Tuberculosis (TB) is a global public health problem; it constitutes one of the most common causes of death (among the first 10 causes). In general terms, TB is the main cause of death due to a single microorganism, *Mycobacterium tuberculosis* (*M. tuberculosis*). In 2021, the World Health Organization (WHO) reported approximately 10.6 million new cases and 1.56 million deaths from TB (global TB report). The incidence of extrapulmonary TB is around 20%. The region with the most reported cases was Southeast Asia with 45%. The region of the Americas reported 2.9% of cases. In Mexico, the number of new TB cases in 2021 was 32,000 with 4800 deaths; the reports of cases of neurotuberculosis are scarce and with a poor analysis [1,2].

Social inequality, demographic factors such as the age of the population, human migration, as well as the increase in the prevalence of people with HIV infection have been identified as the most important causes of the current serious situation of tuberculosis in the world. The different presentations of TB of the central nervous system, intracranial and intraspinal neuro-TB are responsible for 5 to 10% of all extrapulmonary tuberculosis. However, due to the difficulty in diagnosis and the existence of underreporting, these data could be underestimated [3,4,5].

The most common manifestation of intracranial neuroTB is tuberculous meningitis, which can develop insidiously or abruptly; it can be complicated by hydrocephalus, stroke, arachnoiditis, tuberculoma or cranial neuropathy, while intraspinal neuroTB has a chronic course with three main clinical features: cold abscesses, neurological deficit and kyphotic spinal deformity [4]. The diagnosis of intracranial and intraspinal neuroTB requires high suspicion due to its paucibacillary behavior; several patients are diagnosed in advanced stages of the disease; neurological complications lead to disability and fatal outcome in productive stages of life [5,6].

Spinal TB, known as “Pott’s disease” (since 1779), begins with a primary infection at the pulmonary level and spreads through the hematogenous route to an extrapulmonary focus (among others lymphadenitis, renal, cutaneous, miliary and skeletal system). *M. tuberculosis* spreads through the anterior and posterior spinal arteries of each vertebra, as well as through the valves of the Batson’s paravertebral venous plexus [7,8]. Park et al. (2023), present the following types of involvement in intraspinal neuroTB: spondylitis, spondylodiscitis, myelitis, arachnoiditis, intramedullary tuberculoma and longitudinally extensive transverse myelitis [9]. Regarding spondylitis/spondylodiscitis, which can clinically present as back pain, weakness in the lower limbs and paraplegia; by imaging, the involvement of three or more vertebral bodies can be observed with little involvement of the intervertebral disc. Bone marrow edema with contrast enhancement (early phase). Paravertebral or paraspinal abscess and vertebral body collapse [9,10]. The time from the onset of symptoms to the time of diagnosis in intraspinal neuroTB can range from 4 to 6 months, despite advances in imaging tools, diagnostic delay constituting a determining factor for a worse diagnosis, presentation of neurological deficit and an increase in the likelihood of using surgical management [7].

Intracranial neuroTB begins in the lungs with extrapulmonary dissemination through hematogenous or lymphatic routes or extension to the meninges or formation of a parameningeal granuloma (Rich’s focus) and may be the gateway, due to granuloma rupture, of the bacillus into space subarachnoid. The release of *M. tuberculosis* bacilli produces an inflammatory response of the host mediated by cytokines that can lead to the presentation of leptomeningitis, optomeningitis, vasculitis, hydrocephalus, cranial neuropathies and tuberculomas, among other presentations [5].

Thakur et al. (2018) explains that in meningeal TB, three stages or prodromal phases can be observed. The first, which usually lasts for 2 or 3 weeks, is characterized by the insidious appearance of malaise, headache, low-grade fever and confusion. The second stage of the disease (meningitic phase) presents with more pronounced neurological features, including meningismus, prolonged headache, lethargy and cranial nerve clinical findings. In the third phase, stupor, coma and seizures are observed. Death can occur, in most untreated patients, within 5 to 8 weeks of illness onset [3].

Park et al. (2023), classifies the following types of involvement in intracranial neuroTB: leptomeningitis, vasculitis, tuberculoma, abscess, cerebritis, miliary TB, pachymeningitis and ventriculitis and as complications of intracranial neuroTB: hydrocephalus, acute infarction and cranial nerve palsy [9].

Tuberculosis in Mexico is an endemic disease, underreported, unsuspected and neglected disease in our population. This is observed through a lag in the governing standards for its prevention, detection and treatment. It becomes more complex in the case of extrapulmonary tuberculosis, because it affects our hospital services, since patients with long clinical evolution are referred to our hospital, without diagnosis and therefore without treatment, with irreversible neurological damage and a poor prognosis. In our study, we were able to observe and analyze the clinical, imaging and outcome characteristics of a significant number of patients with intraspinal and intracranial neurological complications through a retrospective cohort over 22 years (2000–2022). For this reason, we hope that our work provides useful data not only to improve our daily clinical practice, but also to contribute to national and international information, as well as to the comparison with other countries with similar characteristics to ours.

## 2. Materials and Methods

### 2.1. Study Design and Population

A retrospective cohort study of hospitalized patients with diagnosis of intraspinal and intracranial neuroTB was designed in a hospital in Western Mexico. The Institutional Review Board approved the study under registration numbers R-2020-1301-075 and R-2020-1301-120. Medical records of patients with neurotuberculosis were reviewed over a period of 22 years (2000–2022). The inclusion criteria were: indistinct sex and age ≥ 18 years. Patients with incomplete records were excluded. Also excluded were: patients with spinal TB without neurological involvement, as well as patients with isolation of microorganisms other than the *M. tuberculosis* complex and with positive serology for *Brucella* spp. To contrast our findings of intraspinal neuroTB, we mainly used the available literature on spinal TB. For the diagnosis of intraspinal tuberculosis, the 2015 Infectious Diseases Society of America (IDSA) Guidelines and the 2013 Duarte algorithms were used [11,12]. The Lancet score was used to define the diagnosis of intracranial tuberculosis [13]. Additionally, the severity of meningitis is classified using the criteria of the Medical Research Council (MRC) [14,15].

### 2.2. Data Collection and Diagnostic Methods

The clinical and paraclinical records of hospitalized patients attended by the Traumatology and Orthopedics, Neurosurgery, Neurology and Infectious Diseases services for 22 years were collected and reviewed. Patients with a diagnosis of intraspinal and intracranial neuroTB were selected. The patients were monitored either in the outpatient clinic or during hospitalization episodes. Study variables included demographic data, history of tuberculosis (previous or concomitant), associated neurological complications, symptoms, comorbidities and outcomes. Diagnosis of tuberculosis in fluids or tissues included at least one of the following: smear of acid-fast bacilli (AFB), detection and isolation of *M. tuberculosis* by Löwenstein–Jensen. Nucleic acid amplification tests. Automated molecular tests included GeneXpert MTB/RIF real-time PCR. Histopathological studies of biopsy of brain, vertebral or intervertebral disc tissue with findings of epithelioid granulomas, caseous necrosis and Langerhans giant cells. Enhanced magnetic resonance imaging (MRI) and enhanced computed tomography (CT) were the most common methods for detecting tuberculoma, leptomeningitis, stroke, hydrocephalus, spondylodiscitis, abnormalities of the spine, pre/paravertebral or epidural vertebral abscess [8,9,10,16,17].

The following classification (adapted Garg et al. criteria, 2022) [18] was used to describe the imaging findings of spinal TB [18]:

Number of levels involved:

- One level: With paradiscal morphology, when a complex is affected (vertebra-disc-vertebra). If the morphology is central or appendiceal (2 contiguous vertebrae involved).
- Two levels: With paradiscal morphology when there are two complexes (each complex formed by a disc with 2 vertebrae involved). If the morphology is central or appendiceal (2 complexes, each complex formed by 2 contiguous vertebrae involved).
- Three or more levels: For paradiscal morphology when there are 3 or more complexes (vertebra–intervertebral disc–vertebra). For central or appendiceal morphology, there would be 3 or more complexes of 2 contiguous vertebral involved.
- Continuous multilevel involvement: patients with involvement of ≥3 continuous paradiscal, central, or appendiceal levels.
- Non-contiguous multifocal involvement/skipped lesion: Patients with involvement of ≥3 non-contiguous or skipped lesions of any morphology (paradiscal, central, or appendiceal) [18].

In this research work we used the definitions of Batirel et al. (2015) [19], who define sequelae as any pathological condition that is a consequence of neuroTB, which includes: neurological motor deficit (paraparesis/plegia), compression of nerve roots or spinal deformity (kyphosis). Unfavorable evolution: Patient who presents sequelae or dies from tuberculosis. Spondylodiscitis: infection of the intervertebral disc and the adjacent vertebral body. Mortality: death from neuroTB [19].

### 2.3. Statistic Analysis

The demographic characteristics, history of tuberculosis, clinical, laboratory, histopathological and imaging findings and complications observed in the neurological cohort were described for intraspinal and intracranial neuroTB. Likewise, the duration of treatment, and outcomes (favorable and unfavorable), including mortality in patients with intracranial neuroTB. A risk analysis was performed, using univariate analysis, among patients with intraspinal and intracranial neuroTB, exclusively to compare demographic characteristics (age, sex and occupation), comorbidities, concomitant diseases, clinical and laboratory findings and outcomes. Two separate groups were formed for analysis: the first group consisting of patients diagnosed with intraspinal neuroTB and the second group of patients with intracranial neuroTB. Also using univariate analysis, risk factors for outcomes (favorable and unfavorable) in patients presenting with intraspinal neuroTB were calculated; and risk factors for outcomes in patients with intracranial neuroTB and a univariate analysis of mortality was also performed. The univariate analysis, to calculate the risk factors, was performed using the Mann–Whitney U test. Qualitative variables were examined using the χ2 or Fisher’s exact test. Results were considered statistically significant when *p* < 0.05. SPSS V25 ^®^ (IBM, Armonk, NY, USA) was used for statistical analysis. The diagnostic delay in relation to the onset of the patients’ symptoms, referred to in months, was analyzed. Additionally, from our gallery, images and a description of a representative clinical case of patient with intraspinal neuroTB and another of intracranial neuroTB are presented.

## 3. Results

**Figure 1:**
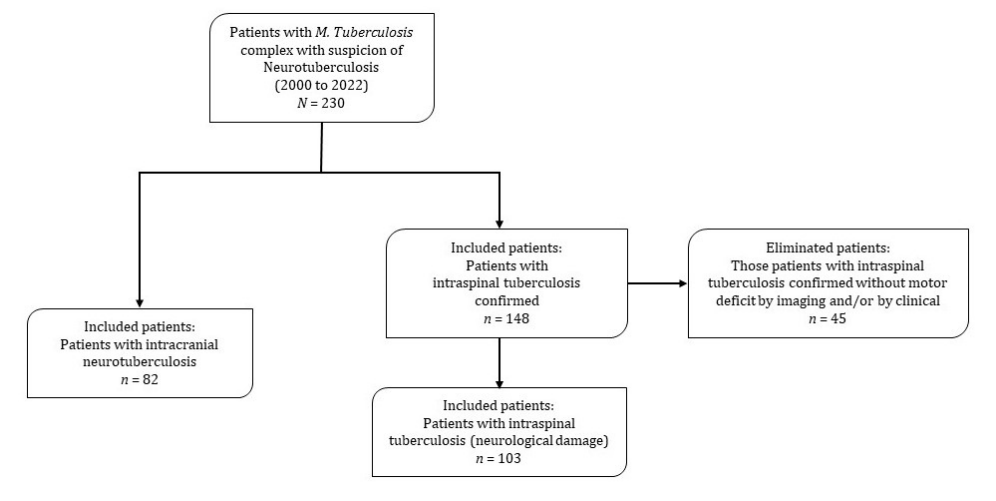
Flow diagram of the intracranial and intraspinal neurotuberculosis cohort.

In the period of time considered in the cohort (22 years), 230 records of patients with diagnosis of tuberculosis and a high probability of having neuroTB were reviewed in depth. Of 148 patients who presented vertebral tuberculosis, 45 patients were excluded because they did not present neurological damage, ruled out by imaging and clinical data. The frequency of intraspinal neuroTB was estimated out of 69.6% (103/148) in hospitalized patients with Pott’s disease. Therefore, the neurological cohort was made up of a total of 185 patients: 103 (55.7%) patients with intraspinal neuroTB and 82 (44.3%) patients with intracranial neuroTB. It should be noted that seven patients presented both intracranial and intraspinal neuroTB and were classified in the group of patients with intracranial neuroTB. This decision to assign them to this group was taking into account that intracranial neuroTB has a poorer prognosis (Figure 1).

**Figure 2:**
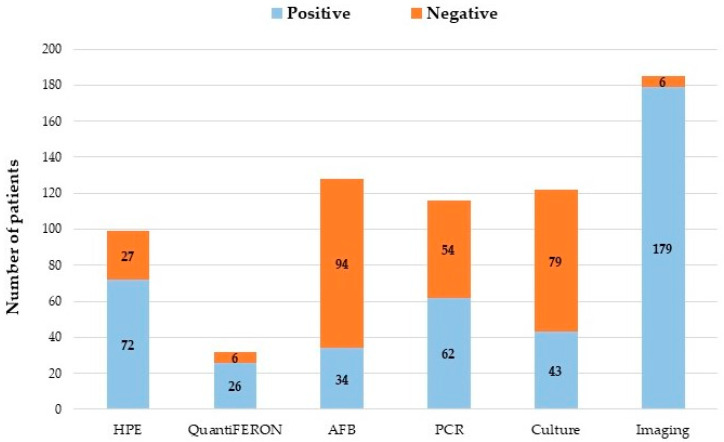
Diagnostic tools in the cohort of patients with intraspinal and intracranial neurotuberculosis. HPE: Histopathological study. AFB: Acid-fast bacilli. PCR: Polymerase chain reaction. Imaging: Magnetic resonance and computed tomography.

Figure 2 graphically presents the different diagnostic tools used and the results in the diagnosis of tuberculosis of the patients included in the cohort. Additionally, imaging (nuclear magnetic resonance and computed tomography) is presented given its importance in the early detection of complications of neuroTB and discrimination of other pathologies.

The group of patients diagnosed with intraspinal neuroTB was represented by 62 (60.2%) men, with a median age of 53 years (IQR 39–62 years): 78 patients (75.7%) were older than 40 years. In the initial assessment (admission), the patients in this group presented the following comorbidities: diabetes mellitus (DM), immunocompromised (due to HIV infection, DM, chronic kidney disease or cancer), immunosuppression (caused by drugs that produce systemic immunosuppression such as corticosteroids and anti-tumor necrosis factor alpha), chronic kidney disease (CKD), kidney transplant, HIV infection and history of tuberculosis (Table 1).

These patients were diagnosed concomitantly with other tuberculosis, such as: vertebral osteomyelitis, pulmonary tuberculosis, disseminated tuberculosis, pleural/empyema, extraspinal osteomyelitis, at the pericardial or mediastinal level, vertebra-cutaneous sinus pathways and gastrointestinal–peritoneal tuberculosis and genitourinary tuberculosis. Vertebral osteomyelitis was found in 101 patients (98.1%) and extraspinal osteomyelitis in 11 patients (rib, hip, sternum and knee). Pulmonary tuberculosis was observed in 21 patients (20.4%). Pain in the spine (back/neck) was present in 102 patients (99%), fever in 44/97 patients (45.4%), weight loss in 39/96 (40.6%), paraparesis/paraplegia was found in 103 (100.0%) and lymphopenia ≤ 0.900 cells (10^3^/µL) in 14/64 patients (21.9%), (Table 1).

A delay of ≥5 months (150 days) to make a diagnosis was observed in 68 patients (66.0%) with intraspinal neuroTB. The duration of treatment for patients with intraspinal neuroTB was estimated at 12 months (IQR 12–14 months) (Table 1).

**Figure 3:**
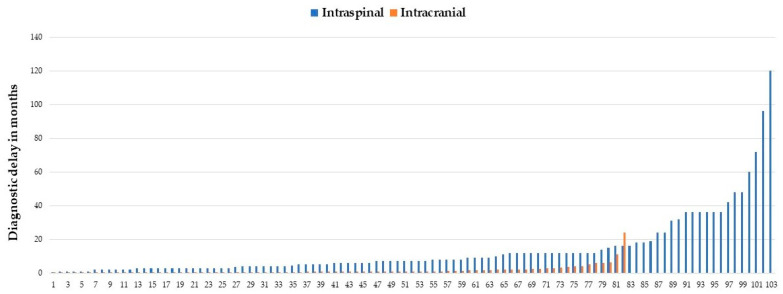
Each case of intraspinal and intracranial neurotuberculosis.

Figure 3 provides information on the delay in diagnosis for each of the patients included in the groups with intraspinal (103 patients) and intracranial (82 patients) neuroTB. Where longer delay in diagnosis were observed for patients with intraspinal neuroTB, reaching up to 120 months.

Of the 103 patients included in the intraspinal neuroTB group, 4 died from causes other than tuberculosis. Of the remaining 99 patients, 34 patients (34.3%) had favorable events (with clinical and imaging improvement) and unfavorable events (death due to TB, or without clinical and imaging improvement), 65 patients (65.7%). Of the three patients who died due to TB, two of them occurred in the first ≤2 months of treatment (Table 1).

Of the 82 patients diagnosed with intracranial neuroTB, 53 (64.6%) were men. Patients in this group had an age range of 17–80 years and a median of 41 years (IQR 29–50 years). The comorbidities presented by this group of patients were: DM 15/81 (18.5%), immunocompromised 52/78 (66.7%), immunosuppressed 44/72 (61.1%), kidney transplant 10 patients (12.2%), HIV infection 22/79 (27.8%) and a history of tuberculosis in 19 patients (23.2%).

Concomitant tuberculosis was found in this group of patients: pulmonary 28 (34.1%), disseminated tuberculosis 34 (41.5%), gastrointestinal 6 patients (7.3%), intraspinal neuroTB 7 patients (8.5%) and genitourinary 5 patients (6.1%), among others. Fever was present in 69/81 patients (85.2%), weight loss in 47/77 (61.0%), hemiparesis or paraparesis in 8 patients (9.8%) and lymphopenia ≤ 0.900 cells (10^3^/µL) in 33/51 patients (64.7%). The median in the diagnostic delay was estimated at 21 days (IQR 13–48 days). Fifty-eight (70.7%) patients were diagnosed in the first 40 days of evolution. The duration of anti-tuberculosis treatment was estimated to be a mean of 12 months with an IQR of 3 to 12 months.

Of the patients with intracranial neuroTB, 32/81 (39.5%) had a favorable evolution (with clinical and imaging improvement) and an unfavorable 49/81 (with neurological sequelae or death). Of these 5/10 kidney transplant patients (50%) lost the graft. Hospital mortality of patients with intracranial neuroTB was estimated at 29.6% (24/81). Died during the first 2 months of treatment 20/81 patients (24.7%) (Table 1).

Contrasting demographic characteristics, comorbidities, clinical and laboratory findings, treatment time and patient outcomes in the neurotuberculosis cohort, the following were found to be significant risk factors for patients with intracranial neuroTB: age ≤ 30 years (OR 2.9, CI 1.6–6.1, *p* = 0.005), white-collar workers (OR 9.9, CI 2.1–46.4, *p* = 0.001), immunocompromised (OR 2.0, CI 1.1–3.7, *p* = 0.026), immunosuppressed (OR 4.6, CI 2.4–9.1, *p* = 0.000), kidney transplant (OR 7.0, CI 1.5–32.9, *p* = 0.006) and HIV infection OR 9.6, CI 3.1–29.1, *p* = 0.000). Present disseminated tuberculosis (OR 2.8, CI 1.4–5.3, *p* = 0.002), and clinical symptoms of fever (OR 6.9, CI 3.3–14.4, *p* = 0.000) and weight loss (OR 2.3, CI 1.2–4.2, *p* = 0.008). In the laboratory findings, lymphopenia (OR 6.5, CI 2.9–14.9, *p* = 0.000). As well as a higher risk of death for patients with intracranial neuroTB (OR 13.5, CI 3.9–46.8, *p* = 0.000) (Table 1).

### 3.1. Intraspinal NeuroTB

Considering the imaging findings of intraspinal neuroTB, 101/103 patients (98.1%) presented spondylodiscitis, one with central spondylitis (without discitis) and another with intramedullary tuberculoma. Also in the imaging findings, the regional distribution of vertebral involvement was as follows: cervical 5/102 (4.9%), dorsal 29/102 (28.4%), dorsal–lumbar 4/102 (3.9%), lumbar 25/102 (24.5%), lumbo–sacral 8/102 (7.8%) and multilevel lesions 31/102 (30.4%). Continuing with the description of imaging, we found the following findings in relation to the number of levels involved in patients with intraspinal neuroTB: 1 level 70/102 (68.6%), 2 levels 16/102 (15.7%), ≥3 levels 16/102 (15.7%), contiguous multilevel lesions 27/102 (26.5%), non-contiguous multilevel segments/skip lesions 10/102 (9.8%). Regarding the pattern of vertebral involvement, the following was found: paradiscal 101/102 patients (99.0%), central (C7 to T3/L5) 1/102 (0.98%) and appendiceal 5/102 (4.9%) (Table 2).

All of the patients included in this group on admission presented motor deficit, spinal instability 95/101 (94.1%), pre/paravertebral abscess 86/102 (84.3%), destruction vertebral 80/102 (78.4%), epidural abscess 79/102 (77.5%) and myelopathy 71/102 (69.6%). These patients also presented other complications such as: destruction as vertebra plana, psoas abscess, wedge fracture, kyphosis, intraosseous abscesses, spinal arachnoiditis, complete spinal cord syndrome and intramedullary tuberculoma (Table 2).

According to the univariate analysis, the following risk factors for unfavorable outcomes were found: age older than 40 years (OR 3.0, CI 1.2–7.8, *p* = 0.019) and having DM (OR 3.4, CI 1.3–8.9, *p* = 0.011). Late diagnosis, calculated at ≥150 days, was also observed as a risk factor (OR 2.8, CI 1.2–6.7, *p* = 0.018). From the imaging findings, it was observed as a risk factor for unfavorable outcomes having ≥2 levels involved (OR 5.2, CI 1.6–16.4, *p* = 0.003), ≥3 levels involved (OR 8.8, CI 1.1–70.1, *p* = 0.017), multilevel involvement (OR 7.1, CI 2.0–25.7, *p* = 0.001) and having a contiguous multilevel (OR 5.5, CI 1.5–19.9, *p* = 0.007) (Table 3).

Among the pre-treatment (medical-surgical) findings or complications, it was found that motor deficit was present in the total group (99 patients) of intraspinal neuroTB; and post-treatment was observed in patients with a favorable outcome 46/99 (46.5%). While only one patient of those who had a favorable evolution was left with motor deficit but with improvement (paraparesis).

In patients with unfavorable results before treatment, the following risks were also observed: presenting kyphosis was a risk factor for patients to have unfavorable outcomes (OR 7.5, CI 1.6–34.4, *p* = 0.004); vertebral destruction ≥ 2 levels (OR 4.8, CI 4.6–14.0, *p* = 0.003); ≥3 levels (OR 12.3, CI 1.6–96.5, *p* = 0.003) and vertebral destruction ≥ 3 vertebrae (OR 6.4, CI 1.4–29.5, *p* = 0.009) (Table 3).

In patients with unfavorable outcomes, were found as risk factor post-surgical instability 26/64 (40.6%) (OR 6.8, CI 1.9–24.8, *p* = 0.001). Likewise, in those patients with residual abscess, or due to lack of release of the spinal cord by anterior surgical approach, they presented a risk of unfavorable results in the analysis (OR 3.4, IC 1.4–8.3, *p* = 0.005), (Table 3).

**Figure 4:**
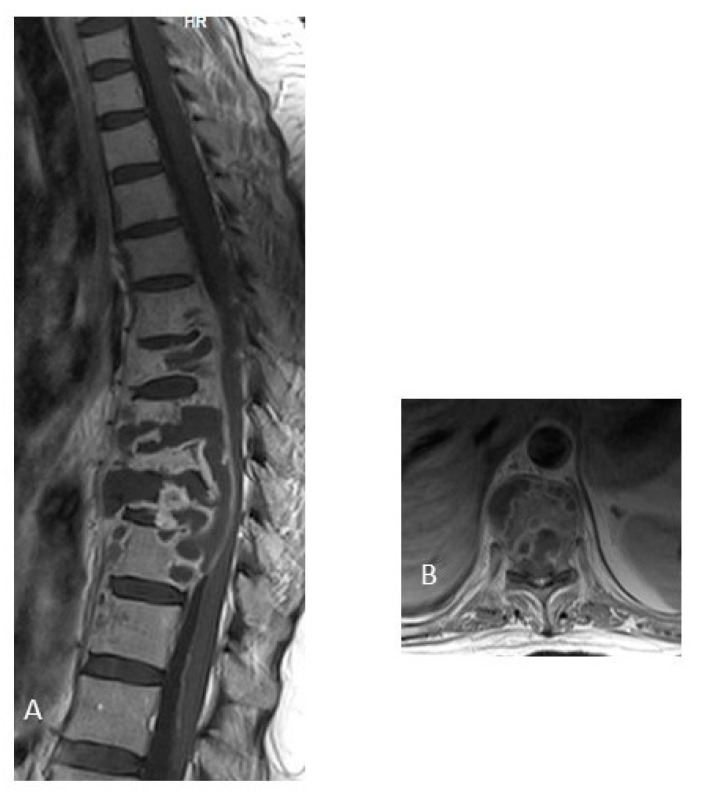
MRI of thoracic spine post-contrast T1-weighted image. (**A**) Sagittal image, and (**B**) Axial image, with lytic lesions of the vertebral bodies and appendiceal involvement from T7 to T9, with prevertebral and epidural abscess. A 29-year-old woman, with pulmonary tuberculosis, who had low back pain for 14 months, spinal tissue culture positive for *M. tuberculosis*. The image shows multilevel tuberculous spondylodiscitis from T5 to T11 with disc-vertebral destruction with epidural abscess with compression of the spinal cord from T7 to T10. (The photo is from our image gallery).

### 3.2. Intracranial Neurotuberculosis

According to the criteria proposed by Marais et al. (2010), were classified as having definitive meningeal tuberculosis or Lancet I, 41 patients (50.0%), as probable meningeal tuberculosis or Lancet II, 28 patients (34.1%) and as possible meningeal tuberculosis or Lancet III, 13 patients (15.9%) [13] (Table 2).

Three patients had tuberculomas without meningitis or with normal cerebrospinal fluid. Thus, 79/82 (96.3%) patients with meningitis were classified, of whom, according to the MRC meningitis severity criteria: 7 patients (8.9%) presented Grade I meningitis, 43 patients (54.4%) presented Grade II meningitis and 29 (36.7%) Grade III meningitis (Table 2).

The following complications were found in the patients who had meningitis: tuberculomas, cranial neuropathy, hydrocephalus, leptomeningitis, cerebral infarction, hydrocephalus with tuberculomas, hydrocephalus with cerebral infarction and tuberculous abscess. Some patients presented more than one of these complications (Table 2).

Cranial neuropathy associated with meningitis was observed in 33/82 patients (40.2%). Table 2 shows the cranial nerves that were involved in these patients. Sixth cranial nerve palsy occurred more frequently, in 17/33 (51.5%) of the patients. It should be noted that some patients had more than one affected cranial nerve (Table 2).

One patient died of causes unrelated to tuberculosis and was therefore excluded from the intracranial cohort to calculate risk factors in the univariate analysis. According to the univariate analysis performed among patients with favorable and unfavorable outcomes, age > 40 years was found as a risk factor (OR 3.0, CI 1.2–7.6, *p* = 0.018), and a diagnostic delay of ≥24 days (OR 2.7, CI 1.1–6.9, *p* = 0.035).

Among the clinical findings that were observed as risk factors for unfavorable outcomes the following: hemiparesis (OR 10.9, CI 2.9–40.7, *p* = 0.000), drowsiness (OR 5.8, CI 2.0–16.6, *p* = 0.001), stiff neck (OR 4.5 CI 1.6–12.9, *p* = 0.003), alterations in consciousness (OR 3.4, CI 1.3–9.2, *p* = 0.012) and alterations in language (OR 2.9, CI 1.1–7.2, *p* = 0.023).

Among the laboratory findings, lymphopenia ≤ 0.900 cells (10^3^/µL) were observed as a risk factor (OR 8.0, CI 2.1–31.0, *p* = 0.003). Those patients who had definitive meningeal tuberculosis (Lancet I) had a higher risk of an unfavorable outcome (OR 8.1, CI 2.9–22.8, *p* = 0.000). Of the complications associated with meningitis, risk factors for unfavorable outcomes were found: cranial neuropathy (OR 4.0, CI 1.5–11.1, *p* = 0.005) and hydrocephalus (OR 4.1, CI 1.3–12.3, *p* = 0.014) (Table 4).

**Figure 5:**
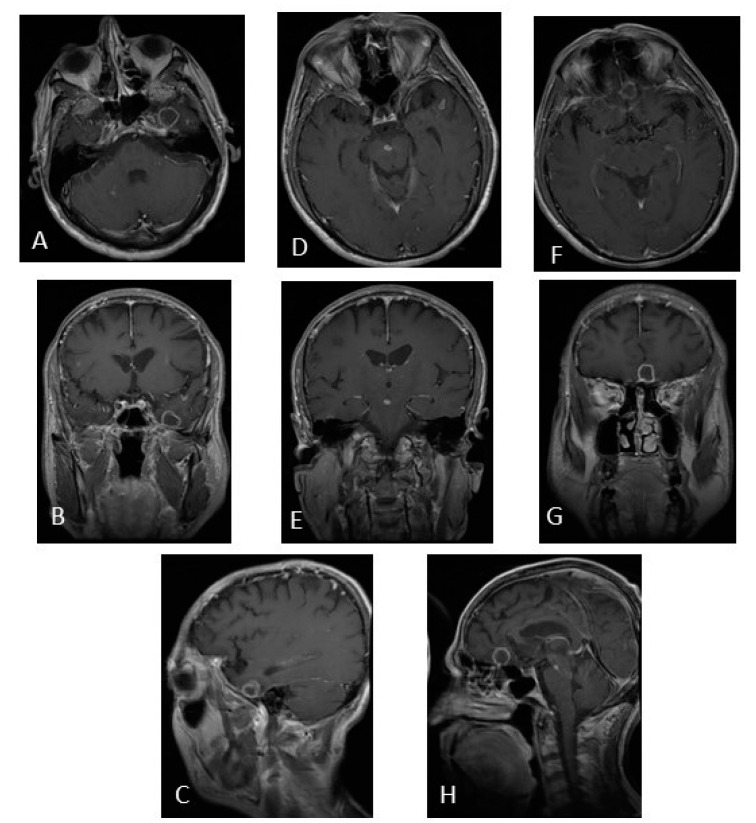
MRI of brain post-contrast T1-weighted image. (**A**–**C**) Tuberculoma in the left temporal lobe. (**D**,**E**) Tuberculoma in the midbrain. (**F**–**H**) Tuberculoma in the frontal lobe. Sixty-four-year-old patient, with previous use of infliximab, with a fever of 6 months of evolution. Clinical improvement at 2 weeks, and imaging improvement at 9 months after starting antituberculous drugs. (The photo is from our image gallery).

### 3.3. Mortality

Twenty-four patients died due to intracranial neuroTB 24/81 (29.6%), of which 20 (83.3%) received ≤2 months of anti-tuberculosis treatment. Ten patients had HIV infection and four had a kidney transplant (Table 4).

Table 5 shows the risk factors found to be associated with mortality for intracranial neuroTB. The following were found as risk factor associated: among the comorbidities, HIV infection (OR 3.8, CI 1.3–11.2, *p* = 0.012) and having concomitant tuberculosis such as: disseminated tuberculosis (OR 3.6, CI 1.3–9.8, *p* = 0.010), gastrointestinal and peritoneal tuberculosis (OR 14.7, CI 1.6–134, *p* = 0.008).

Among the clinical characteristics were found as risk factors: language alteration (OR 5.2, CI 1.7–15.9, *p* = 0.003) consciousness alterations (OR 16.7, CI 2.1–132.6, *p* = 0.000), hemiparesis (OR 3.0, CI 1.1–8.1, *p* = 0.025) and drowsiness (OR 15.4, CI 4.5–52.6, *p* = 0.000).

In the laboratory findings, lymphopenia ≤ 0.900 cells (10^3^/µL) were found as a risk factor for mortality (OR 11.5, CI 2.3–59, *p* = 0.001).

Patients with intracranial neuroTB classified with the Lancet I score (OR 8.6, IC 2.6–28.5, *p* = 0.000) were found to have a risk factor for mortality.

Among the complications associated with meningitis, the following were found as risk factors for mortality: cerebral infarction (OR 7.9, CI 2.1–29.5, *p* = 0.002) and hydrocephalus (OR 4.0, CI 1.5–11.0, *p* = 0.006); having cerebral infarction plus hydrocephalus (OR 14.7, CI 1.6–134, *p* = 0.008) (Table 5).

## 4. Discussion

The objective of this study was to analyze the demographic, clinical, imaging and evolution characteristics of patients with neurological complications secondary to tuberculosis from a retrospective cohort from 2000 to 2022, of hospitalized patients diagnosed with intraspinal and intracranial neuroTB in Western Mexico hospital.

In the study period in our hospital, there was 3% mortality due to intraspinal neuroTB, similar to that reported by Batirel et al. (2015) [19] of 2.2% in a multinational and multicenter study [19]. On the other hand, as expected, a much higher mortality was observed for patients with intracranial neuroTB estimated at 29.6%, similar to that found by Modi et al. (2017) reported in 25.4% and found lower than that reported by Cantier et al. (2018), (43%) in patients from a multicenter cohort [20,21].

In our hospital, routinely, the culture of samples obtained from the different organs or target tissues is used, specifically Löwenstein–Jensen. AFB test is also routinely performed and real-time PCR for tuberculosis is also available. Histopathological findings are made with an intentional search for tuberculosis. Confirmation of tuberculosis positivity was obtained in all patients included in the study cohort. However, MRI was an important tool, initially to suspect tuberculosis and also to locate the lesions and classify the damage, both in patients with intraspinal and intracranial neuroTB. Several studies highlight the usefulness of MRI in the early detection of complications of neuroTB, given the low sensitivity of cultures and AFB; in addition, given the paucibacillary nature of *M. tuberculosis* and the challenge that this entails in the handling of extrapulmonary samples, which makes diagnosis through microbiology difficult [5,7,22,23,24]. Several criteria have been developed in recent years for the diagnosis of intraspinal neuroTB, and the importance of magnetic resonance imaging continually increases the sensitivity of these criteria and allows initiation of treatment in neuroTB even in the absence of confirmation [25].

### 4.1. Intraspinal NeuroTB

All the patients that were included in the intraspinal group had neurological damage, since it was a specific inclusion criterion to consider that they had neuroTB, 45/148 patients with spinal TB without neurological damage were excluded. The frequency of intraspinal neuroTB was estimated out of 69.6% (103/148) in hospitalized patients with spinal TB disease. In a multicenter retrospective study from China, which included 1378 cases of hospitalized patients with spinal TB (2007–2016), almost half (49.9%) of the patients presented neurological damage [26].

The delay in diagnosis was greater for patients with intraspinal neuroTB (5 months), reaching up to 120 months, and it was considered as a risk factor for presenting an unfavorable event in our study. Garg et al. (2022) [18], report a delay in diagnosis of 4.5 months between symptom onset and diagnosis. The variety of clinical manifestations, as well as the presentation of very non-specific symptoms, constitutes a challenge to provide a timely and correct diagnosis. In this way, the delay in diagnosis (4 to 6 months) could be the factor that contributes the most to a worse prognosis and puts the patient at risk of presenting neurological deficits and having a greater probability of requiring surgical treatment [18]. Furthermore, once symptoms progress to neurological deficits, a significant number of patients may never recover their neurological function [7].

The group of patients diagnosed with intraspinal neuroTB was over 40 years old (75.7%), had a median age of 53 years, and was represented by a greater number of men (60.2%). In a multicenter study of patients hospitalized for intraspinal TB in China, reporting a mean age of 43.7 years and also represented a larger number of men (58.4%) [26]. On a review study on spinal TB, they also report 53% of male patients with a median of 43 years. That agrees with the demographic data obtained in our study, for patients with intraspinal neuroTB. Among the demographic variables, old age has been reported as a risk factor for an unfavorable outcome; we found age over 40 years also as a risk factor [19,26,27,28].

In our study, all patients included with intraspinal neuroTB on admission presented motor deficit mainly due to: spinal instability, pre/paravertebral abscess, vertebral destruction, epidural abscess and myelopathy; as well as spondylodiscitis. The main symptoms of patients with intraspinal neuroTB were: spinal pain (back/neck) present in 99% of patients, fever in 45.4% and weight loss in 40.6% and paraparesis/paraplegia was found in all patients; these symptoms are reported as the most common for spinal tuberculosis, in a review article, where they are reported with a lower percentage: back pain 70.4%, fever 32.7% and loss of body weight 30.3%. In addition to considering that paraplegia is the most devastating complication in spinal TB [28].

According to the univariate analysis, as a risk factor for unfavorable results: presenting kyphosis, having involvement of two or more levels and three or more levels, as well as presenting involvement at various levels and having involvement contiguous multilevel. This result is consistent with the fact that vertebral destruction of three or more vertebrae was also found as a risk factor for unfavorable results. Almost in its entirety 99.0%, with a paradiscal pattern of vertebral involvement. In an epidemiological study that included 1652 patients with spinal tuberculosis, they found 81% of patients with one level of involvement, 12% with two and 7% with three or more levels of involvement; and 82% paradiscal involvement [18]. Furthermore, it is reported that the poor prognosis for the progression of the deformity is influenced by the severity of the angulation of the kyphosis, where the kyphosis is >60 degrees with more than three involved vertebrae (where the duration of the disease is greater than 24 months) [16].

### 4.2. Intracranial NeuroTB

Of the group with intracranial neuroTB, 79/82 (96.3%) patients with meningitis were classified. Half of these patients presented, according to the Lancet criteria, definitive meningeal tuberculosis; and in 72 of them (91.1%) ≥ Grade II meningitis (MRC meningitis severity criteria). The three remaining patients (3.7%) had tuberculomas without meningitis; with normal CSF (normal cytochemical and negative culture), which is consistent with the publication by Gupta and Munakomi (2023), who reported that these tuberculomas are deep tuberculous granulomatous foci secondary to bacteremia, which can merge into caseous conglomerates without causing meningitis [17].

Finding in these patients with meningitis the following complications (some patients presented more than one of these complications): tuberculomas, cranial neuropathy, hydrocephalus, leptomeningitis, cerebral infarction, hydrocephalus with tuberculomas, hydrocephalus with cerebral infarction and tuberculous abscess. Manyelo et al. (2021), explain that “tuberculous meningitis is the most devastating form of TB and continues to cause high morbidity and mortality” and they estimated “50% of patients dying or suffering neurological sequelae and complications” [29].

Five patients presented spinal arachnoiditis, with partial or total encapsulation of the spinal cord secondary to gelatinous exudate or fibrosis (CSF with hyperproteinorrhachia >1 g/dL), which presupposes a poor prognosis. Another patient presented optochiasmatic arachnoiditis; all of them with unfavorable evolution despite prolonging tuberculosis treatment for up to 18 months, as commented by Gupta and Munakomi (2023) in their review [17].

Among of the comorbidities presented in the group of patients with intracranial neuroTB, HIV infection was found in 27.8% of the patients, 10 of them died (representing almost half of the deaths) and HIV was found to be risk factor mortality in our study. Higher mortality has previously been reported for these patients and found that having meningeal tuberculosis and HIV infection is a risk factor for mortality during hospitalization [30]. Among patients hospitalized for meningeal TB, HIV has been reported to play a major role in the burden of TB and is associated with low-income settings [31].

Ten of the patients with intracranial neuroTB had a kidney transplant, 5/10 (50%) had graft loss, four out of ten (40%) died from tuberculosis and two of them had previously lost the graft also due to tuberculosis. Boubaker et al., 2013, in a retrospective cohort of 491 transplant patients, reported a loss of renal graft due to tuberculosis of 25%, less than that of our patients; and a slightly higher mortality due to tuberculous meningitis estimated at 43.7% [32].

Meningeal tuberculosis has been reported to be more common in young children (≤5 years), but older age (≥60 years) has also been documented as a risk factor. Our hospital cares for adults and our study cohort includes patients with ≥18 years of age. However, we found the age of ≥40 years as a risk factor for unfavorable outcomes [5,29,33].

A significant number of risk factors for an unfavorable outcome between clinical symptoms and complications were found in patients with intracranial neuroTB, consistent with findings on the MRC Meningitis Severity Criteria Scale: hemiparesis, somnolence, impaired consciousness and language impairment. The main complications found in patients with intracranial neuroTB associated with meningitis, risk factors also for unfavorable outcomes as well as cranial neuropathy and hydrocephalus. The same risk factors were associated with mortality. Added to these risk factors associated with mortality was the presentation of cerebral infarction. The possible complications of meningitis have been widely documented, including hydrocephalus, infarction and cranial neuropathies, also found in our patients. In retrospective studies in adults with meningeal tuberculosis, hydrocephalus was an independent factor of poor outcome or mortality, as found by adjusted analyses [33,34]. The ischemic infarction is reported as a common complication, occurring in 20% to 40% of patients with meningitis [35].

Lymphopenia was also found, among the laboratory findings, as a risk factor associated with unfavorable outcomes and mortality, Shridhar et al. (2022) [35], in their case-control study, explains that host immunological factors contribute to the pathogenesis of tuberculous meningitis; finding that the total lymphocyte count, as well as the CD4+ T-cell count, are reported to be lower in tuberculous meningitis [36].

### 4.3. Limitations

Of 122 cultures performed in the total neuroTB study cohort (intraspinal and intracranial), only 43 (35.2%) were positive for *M. tuberculosis*, so we were limited in analyzing resistance to antituberculosis drugs.

The tuberculosis of the central nervous system involves two different anatomical sites, with specific pathophysiological characteristics, complications and outcomes. Therefore, only the demographic data, comorbidities, concomitant tuberculosis, constitutional symptoms and lymphopenia as a laboratory finding, and mortality were compared between patients in the intraspinal and intracranial neuroTB group. In the reviewed literature, few articles include all the neurological manifestations and complications in a single work, which implied a great challenge.

The main contribution of this work, given the concentration nature of the hospital and the collection of data for 22 years, is to have a significant number of patients with neurological complications. However, despite having a study cohort of so many years, given the low frequency of well-documented patients, we were unable to perform a risk analysis adjusted for unfavorable events and mortality. Even so, the clinical and imaging evidence was explanatory of the evolution of our patients during their follow-up (hospital or outpatient).

## 5. Conclusions

Our hospital is a reference center in Western Mexico and a considerable number of patients admitted to said hospital are referred without a diagnosis, referred for requiring surgical management and some with a clinical evolution of many years upon admission (especially in Pott’s disease). In order to establish diagnoses and therefore more opportune treatments (in time and form), our secondary care hospitals require access to new imaging technologies (mainly magnetic resonance) and laboratory new technologies (PCR for TB).

Patients with intraspinal neuroTB were characterized by having a significant number of destroyed vertebrae and multilevel involvement that conditioned their neurological deficit status. Despite their severe clinical condition, improvement was achieved through medical-surgical management in almost half of the patients. We suggest that in patients presenting with spondylodiscitis with neurological deficit, vertebral destruction and involvement of two or more levels, an intentional search for tuberculosis is made, even though the intervertebral disc is not affected.

Patients with intracranial neuroTB were characterized by presenting mostly meningitis, where hydrocephalus and cerebral infarction were risk factors for mortality. The mortality burden in intracranial neuroTB was determined by HIV infections and renal transplantation patients. The criteria proposed by Marais contributed significantly to the diagnosis of patients with intracranial neuroTB.

## Figures and Tables

**Table 1:** Demographic characteristics, comorbidities, clinical and laboratory findings, treatment time and outcomes of patients in the neurotuberculosis cohort.

Demographic Characteristics, Risk Factors and Comorbidities	Intraspinal NeuroTB	Intracranial NeuroTB		
	*n* = 103 (%)	*n* = 82 (%)	OR (95%CI)	*p*
Male gender	62 (60.2)	53 (64.6)		
Mean age (years), median (IQR), range	53 (41–62), (19–79)	41 (29–50), (17–80)		0.000
Age groups (years)	***n* = 103 (%)**	***n* = 82 (%)**		
≤30	13 (12.6)	24 (29.3)	2.9 (1.6–6.1)	0.005
>30	90 (87.4)	58 (70.7)		
>40	78 (75.7)	42 (51.2)		
White-collar workers	2/73 (2.7)	12/55 (21.8)	9.9 (2.1–46.4)	0.001
Blue-collar workers	31/73 (42.5)	26/55 (47.3)		
Diabetes mellitus (DM)	37/100 (37.0)	15/81 (18.5)		
Immunocompromise ^a^	50/100 (50.0)	52/78 (66.7)	2.0 (1.1–3.7)	0.026
Immunosuppression ^b^	22/87 (25.3)	44/72 (61.1)	4.6 (2.4–9.1)	0.000
Chronic kidney disease (CKD)	13 (12.6)	3 (3.7)		
Kidney transplant	2 (1.9)	10 (12.2)	7.0 (1.5–32.9)	0.006
Human Immunodeficiency Virus (HIV) Infection	4 (3.9)	22/79 (27.8)	9.6 (3.1–29.1)	0.000
History of tuberculosis	13 (12.6)	19 (23.2)		
Concomitant tuberculosis				
Vertebral osteomyelitis	101 (98.1)	-		
Pulmonary TB	21 (20.4)	28 (34.1)		
Disseminated tuberculosis (TB)	21 (20.4)	24 (41.5)	2.8 (1.4–5.3)	0.002
Pleural tuberculosis/empyema	16 (15.5)	5 (6.1)		
Extraspinal osteomyelitis *	11 (10.7)	-		
Vertebro-cutaneous sinus tracts	9 (8.7)	-		
TB in the pericardium and mediastinum	7 (6.8)	1 (1.2)		
Gastrointestinal and peritoneal tuberculosis	3 (2.9)	6 (7.3)		
Genitourinary tuberculosis	1 (1.0)	5 (6.1)		
Clinical and laboratory findings				
Fever	44/97 (45.4)	69/81 (85.2)	6.9 (3.3–14.4)	0.000
Weight Loss	39/96 (40.6)	47/77 (61.0)	2.3 (1.2–4.2)	0.008
Pain spine (back/neck)	102 (99.0)	-		
Paraparesis/paraplegia or hemiparesis	103 (100.0)	8 (9.8)		
Lymphopenia ≤ 0.900 cells/µL, n (%)	14/64 (21.9)	33/51 (64.7)	6.5 (2.9–14.9)	0.000
C-reactive protein > 10 (mg/L), n (%)	46/65 (70.8)	-		
Delay in diagnosis				
≤20 days	2 (1.9)	36 (43.9)		
≤40 days	7 (6.8)	58 (70.7)		
≥150 days (≥5 months)	68 (66.0)	6 (7.3)		
Diagnostic delay time (days), median (IQR), range	214 (92–365), (6–3660)	21 (13–48), (6–730)		
Diagnostic delay time (months), median (IQR), range	7.0 (3–12), (0.2–120)	0.7 (0.4–1.6), (0.03–24)		
Duration antituberculous drugs				
Duration of treatment (months), median (IQR)	12 (12–14)	12 (3–12)		
Positive cultures in Löwenstein–Jensen	21/43 (48.8)	22/79 (27.8)		
Resistance to anti-tuberculosis treatment	1/21 (4.8)	6/22 (27.3)		
Isoniazid resistance	1/21 (4.8)	1/22 (4.5)		
Rifampicin resistance	1/21 (4.8)	2/22 (9.1)		
Pyrazinamide resistance	0/21 (0.0)	3/22 (13.6)		
Ethambutol resistance	0/20 (0.0)	1/22 (4.5)		
Outcomes				
Kidney graft loss	0/2 (0.0)	5/10 (50.0)		
Favorable (improvement)	34/99 (34.3)	32/81 (39.5)		
Unfavorable (without improvement or death)	65/99 (65.7)	49/81 (60.5)		
Deaths (due to tuberculosis)	3/99 (3.0)	24/81 (29.6)	13.5 (3.9–46.8)	0.000
Patients who received ≤2 months of treatment for death due to TB	2/99 (2.0)	20/81 (24.7)	15.9 (3.6–70.4)	0.000

^a^ Immunocompromise: Due to HIV, DM, CKD or cancer. ^b^ Immunosuppression: Caused by systemic immunosuppressive drugs (costicosteroids and anti-TNF alpha factor). IQR: Interquartile range. * Patients with extraspinal osteomyelitis: rib, hip, sternum and knee.

**Table 2:** Imaging findings and complications of patients in the neurotuberculosis cohort.

Intraspinal Neurotuberculosis		*n* = 103 (%)		Intracranial Neurotuberculosis			*n* = 82 (%)
Imaging Findings				Lancet Score			
Spondylodiscitis (SD)		101 (98.1)		Definitive meningeal tuberculosis or Lancet I ^a^			41 (50.0)
Regional distribution of vertebral involvement				Probable meningeal tuberculosis or Lancet II			28 (34.1)
	Cervical	5/102 (4.9)		Possible meningeal tuberculosis or Lancet III			13 (15.9)
	Dorsal	29/102 (28.4)					
	Dorso-lumbar	4/102 (3.9)					
	Lumbar	25/102 (24.5)					
	Lumbo-sacral	8/102 (7.8)					
	Multilevel involvement	31/102 (30.4)		Meningeal tuberculosis severity grades MRC ^b^			
Number of levels involved intraspinal neuroTB				Grade I meningitis			7 (8.9)
	1 level involved	70/102 (68.6)		Grade II meningitis			43 (54.4)
	2 level involved	16/102 (15.7)		Grade III meningitis			29 (36.7)
	≥3 levels involved	16/102 (15.7)					
	Contiguous multilevel involvement	27/102 (26.5)					
	Non-contiguous multilevel segments/skipped lesion	10/102 (9.8)					
Pattern of vertebral involvement							
	Paradiscal	101/102 (99.0)		Imaging findings and/or complications			
	Central (C7 to T3/L5)	1/102 (1.0)		Tuberculomas without meningitis or normal CSF			3 (3.7)
	Appendiceal	5/102 (4.9)		Meningitis (M) + tuberculomas			34 (41.5)
Complications and/or findings on admission				M + cranial neuropathy			33 (40.2)
Motor deficit		103 (100.0)			Cranial nerve palsy II	5/33 (15.2)	
Spinal instability		95/101 (94.1)			Cranial nerve palsy III	11/33 (33.3)	
Pre/paravertebral abscess		86/102 (84.3)			Cranial nerve palsy IV	4/33 (12.1)	
Vertebral destruction		80/102 (78.4)			Cranial nerve palsy VI	17/33 (51.5)	
Epidural abscess		79/102 (77.5)			Cranial nerve palsy VII	10/33 (30.3)	
Myelopathy		71/102 (69.6)		M + hydrocephalus			26 (31.7)
Destruction as vertebra plana		38/102 (37.3)			M + hydrocephalus + VPS	20/26 (76.9)	
Psoas abscess		33/102 (32.4)		Leptomeningitis			16 (19.5)
Destruction of vertebral bodies ≥ 2 levels involved		36/102 (35.3)			Optochiasmatic arachnoiditis	1/16 (6.3)	
Destruction of vertebral bodies ≥ 3 levels involved		20/102 (19.6)		M + cerebral infarction			13 (15.9)
No. of levels with vertebral destruction, median (IQR), range		0 (0–1), (0–8)		M + hydrocephalus + tuberculomas			9 (11.0)
No. of destroyed vertebrae, median (IQR), range		2 (1–2), (0–16)		M + cerebral infarction + hydrocephalus			6 (7.3)
Wedge fracture		28/102 (27.5)		M + tuberculous abscess			1 (1.2)
Kyphosis		25/99 (25.3)					
Anterior or posterior longitudinal ligament involvement		15/101 (14.9)		Spinal arachnoiditis			5 (6.1)
Intraosseous abscesses		11/102 (10.8)					
Complete spinal cord syndrome		5 (4.9)					
Spinal arachnoiditis		4/102 (3.9)					
Intramedullary tuberculoma		1 (1.0)					

^a^ Lancet: Score to support the diagnosis of tuberculous meningitis (Marais et al., 2010) [13]. ^b^ MRC: Severity on admission was graded according to the Medical Research Council criteria. IQR: Interquartile range. CSF: Cerebrospinal fluid. VPS: Ventriculo-peritoneal shunt.

**Table 3:** Outcomes, risk factors and diagnostic delay time in patients with intraspinal neurotuberculosis.

Demographic Characteristics, Risk Factors and Comorbidities	Favorable *n* = 34 (%)	Unfavorable *n* = 65 (%)	OR	CI 95%	*p*
Male gender	18 (52.9)	44 (67.7)			0.150
Median age (years), mean ± standard deviation (SD)	45 ± 15	53 ± 13			0.006 ^‡^
>40 years	21 (61.8)	54 (83.1)	3.0	1.2–7.8	0.019
Diabetes mellitus	7 (20.6)	29/62 (46.8)	3.4	1.3–8.9	0.011
Chronic kidney disease	4 (11.8)	9 (13.8)			1.000
Immunocompromise ^a^	14 (41.2)	35/62 (56.5)			0.152
Immunosuppression ^b^	7/30 (23.3)	14/53 (26.4)			0.756
History of tuberculosis	5 (14.7)	8 (12.3)			0.760
Concomitant tuberculosis					
Disseminated tuberculosis (TB)	4 (11.8)	16 (24.6)			0.188
Pulmonary TB	6 (17.6)	15 (23.1)			0.530
Pleural tuberculosis/empyema	2 (5.9)	14 (21.5)			0.05
Extraspinal osteomyelitis	2 (5.9)	8 (12.3)			0.487
Vertebro-cutaneous sinus tracts	1 (2.9)	8 (12.3)			0.159
Clinical findings and diagnostic delay					
Diagnostic delay ≥ 150 days	17 (50.0)	48 (73.8)	2.8	1.2–6.7	0.018
Diagnostic delay time (months), median (IQR), range	4.8 (3–12), (0.2–60)	8 (4–17), (1–120)			0.027 *
Fever	14/32 (43.8)	28/61 (45.9)			0.843
Weight loss	12/32 (37.5)	27/60 (45.0)			0.488
Paraparesis/paraplegia	34 (100.0)	65 (100.0)			-
Imaging findings					
Cervical Spondylodiscitis (SD)	5/33 (15.2)	0 (0.0)			-
Dorsal SD	7/33 (21.2)	19 (29.2)			0.395
Dorso-lumbar SD	1/33 (3.0)	3 (4.6)			1.000
Lumbar SD	13/33 (39.4)	12 (18.5)			NS
Lumbo-sacral SD	4/33 (12.1)	4 (6.2)			0.436
2 levels involved	3/33 (9.1)	13 (20.0)			0.249
≥2 levels involved	4/33 (12.1)	27 (41.5)	5.2	1.6–16.4	0.003
≥3 levels involved	1/33 (3.0)	14 (21.5)	8.8	1.1–70.1	0.017
Multilevel lesions involvement	3/33 (9.1)	27 (41.5)	7.1	2.0–25.7	0.001
Contiguous multilevel involvement	3/33 (9.1)	23 (35.4)	5.5	1.5–19.9	0.007
Non-contiguous multilevel involvement	0 (0.0)	10 (15.4)			-
Paradiscal involvement	33/33 (100.0)	64 (98.5)			-
Central involvement	0/33 (0.0)	1 (1.5)			-
Appendiceal involvement	1/33 (3.0)	3 (4.6)			1.000
Complications and/or findings pre-treatment					
Motor deficit	34 (100.0)	65 (100.0)			-
Pre/paravertebral abscess	27/33 (81.8)	55 (84.6)			0.723
Epidural abscess	23/33 (69.7)	52 (80.0)			0.255
Psoas abscess	14/33 (42.4)	19 (29.2)			0.192
Intraosseous abscesses	2/33 (6.1)	8 (12.3)			0.487
Complete spinal cord syndrome	0 (0.0)	5 (7.7)			-
Spinal arachnoiditis	1/33 (3.0)	3 (4.6)			1.000
Myelopathy	19 (55.9)	49 (75.4)			NS
Anterior or posterior longitudinal ligament involvement	3/33 (9.1)	12/64 (18.8)			0.252
Spinal instability	31/33 (93.9)	60/64 (93.8)			1.000
Kyphosis	2/32 (6.3)	21/63 (33.3)	7.5	1.6–34.4	0.004
Wedge fracture	9/33 (27.3)	18 (27.7)			0.965
Destruction as vertebra plana	9/33 (27.3)	27 (41.5)			0.166
Vertebral destruction	24/33 (72.7)	52 (80.0)			0.415
Vertebral destruction (No. of vertebrae), median (IQR), range	2 (0–2), (0–3)	2 (1–3), (0–16)			0.036 *
Vertebral destruction ≥ 2 levels	5/33 (15.2)	30 (46.2)	4.8	1.6–14.0	0.003
Vertebral destruction ≥ 3 levels	1/33 (3.0)	18 (27.7)	12.3	1.6–96.5	0.003
≥3 destroyed vertebrae	2/33 (6.1)	19 (29.2)	6.4	1.4–29.5	0.009
Peri or post treatment (post operative) complications					
Motor deficit	1 (2.9)	52 (80.0)			0.000
Residual abscess or lack spinal cord release by anterior surgical approach	16 (47.1)	49 (75.4)	3.4	1.4–8.3	0.005
Instrumentation	26/33 (78.8)	47 (72.3)			0.487
Removal of instrumentation	0/33 (0.0)	6/63 (9.5)			-
Post-surgical spinal instability	3/33 (9.1)	26/64 (40.6)	6.8	1.9–24.8	0.001
Duration antituberculous drugs					
Duration of treatment (months), median (IQR), range	12 (12–12)	12 (12–18)			0.712 *
Deaths (due to tuberculosis)	0 (0.0)	3 (4.6)			-

^a^ Immunocompromise: HIV infection, DM, chronic kidney disease or cancer. ^b^ Immunosuppression: Costicosteroids or anti-TNF alpha factor. NS: Not significant. IQR: Interquartile range. ^‡^ Student t-test. * Mann–Whitney U test.

**Table 4:** Outcomes, risk factors and diagnostic delay time in patients with intracranial neurotuberculosis.

Demographic Characteristics, Risk Factors and Comorbidities	Favorable *n* = 32 (%)	Unfavorable *n* = 49 (%)	OR	CI 95%	*p*
Male gender	24 (75.0)	28 (57.1)			0.101
Mean age (years), mean ± (SD)	36.8 ± 16	42.5 ± 14			0.053 ^‡^
>40 years	11 (34.4)	30 (61.2)	3.0	1.2–7.6	0.018
Immunocompromise ^a^	20/31 (64.5)	31/46 (67.4)			0.794
Immunosuppression ^b^	17/27 (63.0)	17/44 (61.4)			0.893
HIV infection	7 (21.9)	14/46 (30.4)			0.402
Diabetes mellitus	3/31 (9.7)	12 (24.5)			0.143
Kidney transplant	6 (18.8)	4 (8.2)			0.182
Chronic kidney disease	2 (6.3)	1 (2.0)			0.559
History of tuberculosis	10 (31.3)	9 (18.4)			0.181
Concomitant tuberculosis					
Disseminated tuberculosis (TB)	11 (34.4)	22 (44.9)			0.346
Genitourinary TB	1 (3.1)	3 (6.1)			1.000
Gastrointestinal and peritoneal TB	1 (3.1)	5 (10.2)			0.395
Pulmonary TB	11 (34.4)	16 (32.7)			0.872
Pleural tuberculosis/empyema	1 (3.1)	4 (8.2)			0.643
Clinical, laboratory, microbiological and histological findings					
Diagnostic delay ≥ 24 days	10 (31.3)	27 (55.1)	2.7	1.1–6.9	0.035
Diagnostic delay time (days), median (IQR)	16 (11–38)	30 (13–58)			0.328 *
Fever	25 (78.1)	43/48 (89.6)			0.206
Weight loss	15/30 (50.0)	31/46 (67.4)			0.129
Hemiparesis	3 (9.4)	26 (53.1)	10.9	2.9–40.7	0.000
Drowsiness	6 (18.8)	28 (57.1)	5.8	2.0–16.6	0.001
Stiff neck	6 (18.8)	25 (51.0)	4.5	1.6–12.9	0.003
Alterations of consciousness	17 (53.1)	39 (79.6)	3.4	1.3–9.2	0.012
Language alterations	12 (37.5)	31 (63.3)	2.9	1.1–7.2	0.023
Total, lymphocytes/mm^3^, (No. lymphocytes), median (IQR)	1.1 (0.8–2.6)	0.5 (0.3–0.9)			0.002 *
Lymphopenia ≤ 0.900 cells (10^3^/µL)	5/15 (33.3)	28/35 (80.0)	8.0	2.1–31.0	0.003
HIV viral load copies/mL Log, median (IQR)	6.1 (3.5–6.4)	5.1 (1.7–5.8)			0.349 *
Helper lymphocytes CD4 cells/mm3, median (IQR)	136 (58–323)	47 (35–73)			0.088 *
Imaging findings and/or complications and Lancet Score					
Definitive meningeal tuberculosis or Lancet I ^c^	7 (21.9)	34 (69.4)	8.1	2.9–22.8	0.000
Tuberculomas without meningitis or normal CSF	3 (9.4)	0 (0.0)			-
Meningitis (M) + cranial neuropathy	7 (21.9)	26 (53.1)	4.0	1.5–11.1	0.005
M + tuberculomas	11 (34.4)	22 (44.9)			0.346
Leptomeningitis	6 (18.8)	10 (20.4)			0.855
M + cerebral infarction	2 (6.3)	11 (22.4)			0.066
M + hydrocephalus	5 (15.6)	21 (42.9)	4.1	1.3–12.3	0.014
M + cerebral infarction and hydrocephalus	0/32 (0.0)	6/49 (12.2)			-
Meningitis + hydrocephalus + tuberculomas	3 (9.4)	6 (12.2)			1.000
Outcomes					
Patients who received ≤2 months of treatment due to death	0 (0.0)	20 (40.8)			-
Deaths due to TB	0 (0.0)	24 (48.9)			-

SD: Standard deviation. ^a^ Immunocompromise: Due to HIV infection, DM, chronic kidney disease or cancer. ^b^ Immunosuppression: Caused by systemic immunosuppressive drugs (costicosteroids or anti-TNF alpha factor). ^c^ Lancet: Score to support the diagnosis of tuberculous meningitis (Marais et al., 2010) [13]. IQR: Interquartile range. ^‡^ Student *t*-test. * Mann–Whitney U test. OR: Odds ratio. CI: Confidence interval. Log: Logarithm.

**Table 5:** Risk factors for mortality from intracranial neurotuberculosis.

	Survivors *n* = 57 (%)	Deaths *n* = 24 (%)	OR	CI 95%	*p*
Demographic characteristics, risk factors and comorbidities					
Male gender	39 (68.4)	13 (54.2)			0.222
Mean age (years), mean (SD)	39 ± 15.1	43 ± 14.5			0.279 *
>40 years	28 (49.1)	13 (54.2)			0.678
Immunocompromise ^a^	34/56 (60.7)	17/21 (81.0)			0.112
Immunosuppression ^b^	29/48 (60.4)	15/23 (65.2)			0.697
HIV infection	11 (19.3)	10/21 (47.6)	3.8	1.3–11.2	0.012
Diabetes mellitus	1/56 (19.6)	4 (16.7)			1.000
Kidney transplant	6 (10.5)	4 (16.7)			0.472
Chronic kidney disease	2 (3.5)	1 (4.2)			1.000
History of tuberculosis	13 (22.8)	6 (25.0)			0.832
Concomitant tuberculosis					
Disseminated TB	18 (31.6)	15 (62.5)	3.6	1.3–9.8	0.010
Genitourinary TB	1 (1.8)	3 (12.5)			0.076
Gastrointestinal and peritoneal TB	1 (1.8)	5 (20.8)	14.7	1.6–134.2	0.008
Pleural TB/empyema	2 (3.5)	3 (12.5)			0.151
Clinical, laboratory, microbiological and histological findings					
Diagnostic delay ≥ 20 days	28 (49.1)	17 (70.8)			0.073
Diagnostic delay time (days), median (IQR), range	16 (12–50), (6–730)	30 (14–56), (6–336)			0.395 *
Diagnostic delay time (months), median (IQR), range	0.5 (0.4–1.7), (0.03–24)	1.0 (0.5–1.9), (0.2–11)			0.335 *
Fever	46 (80.7)	22/23 (95.7)			0.164
Weight loss	29/54 (53.7)	17/22 (77.3)			0.072
Language alterations	24 (42.1)	19 (79.2)	5.2	1.7–15.9	0.003
Alterations of consciousness	33 (57.9)	23 (95.8)	16.7	2.1–132.6	0.000
Stiff neck	18 (31.6)	13 (54.2)			0.056
Hemiparesis	16 (28.1)	13 (54.2)	3.0	1.1–8.1	0.025
Drowsiness	14 (24.6)	20 (83.3)	15.4	4.5–52.6	0.000
Total, lymphocytes/mm^3^, median (IQR)	1.0 (0.5–1.6)	0.3 (0.3–0.5)			0.000 *
Lymphopenia ≤ 0.900 cells (10^3^/µL)	13/28 (46.4)	20/22 (90.9)	11.5	2.3–59.0	0.001
≥50% Mononuclear cells in CSF	31/52 (59.6)	7/17 (41.2)			0.185
CSF glucose ≤ 40 mg/dL	24/44 (54.5)	14/19 (73.7)			0.175
HIV viral load copies/mL Log, mean (SD)	4.7 ± 2.1	4.4 ± 2.1			0.859 *
Helper lymphocytes CD4 cells/mm^3^, mean (SD)	170 ± 170	45 ± 22.2			0.064 *
Imaging findings and/or complications and Lancet Score					
Definitive meningeal tuberculosis or Lancet I ^c^	21 (36.8)	20 (83.3)	8.6	2.6–28.5	0.000
Tuberculomas without meningitis or normal CSF	3 (5.3)	0 (0.0)			-
Meningitis (M) + cranial neuropathy	21 (36.8)	12 (50.0)			0.271
M + tuberculomas	24 (42.1)	9 (37.5)			0.700
Leptomeningitis	14 (24.6)	2 (8.3)			0.130
M + cerebral infarction	4 (7.0)	9 (37.5)	7.9	2.1–29.5	0.002
M + hydrocephalus	13 (22.8)	13 (54.2)	4.0	1.5–11.0	0.006
M + cerebral infarction + hydrocephalus	1 (1.8)	5 (20.8)	14.7	1.6–134	0.008
M + hydrocephalus + tuberculomas	6 (10.5)	3 (12.5)			1.000
Outcomes					
Patients who received ≤2 months of treatment due to death	0	20 (83.3)			-

^a^ Immunocompromise: Due to HIV infection, DM, chronic kidney disease or cancer. ^b^ Immunosuppression: Costicosteroids or anti-TNF alpha factor.^c^ Lancet: Score to support the diagnosis of tuberculous meningitis (Marais et al., 2010) [13]. IQR: Interquartile range. * Mann–Whitney U test. OR: Odds ratio. CI: Confidence interval. SD: Standard deviation. Log: Logarithm. CSF: Cerebrospinal fluid.

## Data Availability

Not applicable.
